# Photorelease of Pyridyl Esters in Organometallic Ru(II) Arene Complexes

**DOI:** 10.3390/molecules20047276

**Published:** 2015-04-21

**Authors:** Abraha Habtemariam, Claudio Garino, Emmanuel Ruggiero, Silvia Alonso-de Castro, Juan C. Mareque-Rivas, Luca Salassa

**Affiliations:** 1 CIC biomaGUNE, Donostia–San Sebastián 20009, Spain; E-Mails: a.habtemariam@warwick.ac.uk (A.H.); eruggiero@cicbiomagune.es (E.R.); salonsodecastro@cicbiomagune.es (S.A.C.); jmareque@cicbiomagune.es (J.C.M.-R.); 2 IKERBASQUE, Basque Foundation for Science, Bilbao 48011, Spain; 3 Department of Chemistry, University of Warwick, Coventry CV4 7AL, UK; 4 Department of Chemistry and NIS Centre of Excellence, University of Turin, Turin 10125, Italy; E-Mail: claudio.garino@unito.it; 5 Kimika Fakultatea, Euskal Herriko Unibertsitatea and Donostia International Physics Center (DIPC), Donostia–San Sebastián 20080, Spain

**Keywords:** photoactivatable complexes, ruthenium, ruthenium arene, PDT, DFT, photochemistry

## Abstract

New Ru(II) arene complexes of formula [(*η*^6^-*p*-cym)Ru(N-N)(X)]^2+^ (where *p*-cym = *para*-cymene, N-N = 2,2'-bipyrimidine (bpm) or 2,2'-bipyridine (bpy) and X = *m/p*-COOMe-Py, **1**–**4**) were synthesised and characterized, including the molecular structure of complexes [(*η*^6^-*p*-cym)Ru(bpy)(*m*-COOMe-Py)]^2+^ (**3**) and [(*η*^6^-*p*-cym)Ru(bpy)(*p*-COOMe-Py)]^2+^ (**4**) by single-crystal X-ray diffraction. Complexes **1**–**4** are stable in the dark in aqueous solution over 48 h and photolysis studies indicate that they can photodissociate the monodentate *m/p*-COOMe-Py ligands selectively with yields lower than 1%. DFT and TD-DFT calculations (B3LYP/LanL2DZ/6-31G**) performed on singlet and triplet states pinpoint a low-energy triplet state as the reactive state responsible for the selective dissociation of the monodentate pyridyl ligands.

## 1. Introduction

Fuelled by the success of photodynamic therapy (PDT) [1], photoactivation of transition metal complexes for application in biology and medicine has gained momentum and several promising families of complexes have been developed in the last few years as alternative PDT agents for the treatment of precancerous and cancerous diseases [2,3]. As in the case of PDT, light activation allows in principle to obtain temporal control on the cellular effects of metal complexes and to localize their action exclusively in the vicinity of the irradiated tissues. Metal complexes could in principle work through different mechanisms of action to those of currently employed photosensitizers [4,5], a fine prospect since the PDT mechanism relies on the presence of oxygen and many solid tumours are hypoxic [6].

Nevertheless, photochemotherapy is not the only field where photoactivatable complexes have been applied and other research areas have benefitted from the development of light-triggerable systems. Examples are metal-based caged compounds for the controlled delivery of neurotransmitters [7,8] (e.g., γ-aminobutyric and glutamic acid) and small active molecules such as NO [9,10] and CO [11] which exert various bioregulative roles in organisms.

Among the various classes of photoactivatable complexes, Ru(II) arene derivatives of the type [(*η*^6^-arene)Ru(N-N)(X)]^2+^ (where for example arene = *para*-cymene and N-N = bidentate chelating ligand) were reported by Sadler and co-workers to be promising systems for their anticancer properties [12] and more recently also for their photochemical features (e.g., N-N = bpm) [13,14]. It was shown that when the monodentate X ligand is a pyridine or a substituted pyridine these complexes are extremely stable in aqueous solution in the dark, but they can be selectively photoreleased upon light excitation. Controlling this process with light holds promise since reactive aqua species as [(*η*^6^-arene)Ru(N-N)(OH_2_)]^2+^ can be generated *in situ*, potentially triggering the biological activity of the complexes.

**Figure 1:**
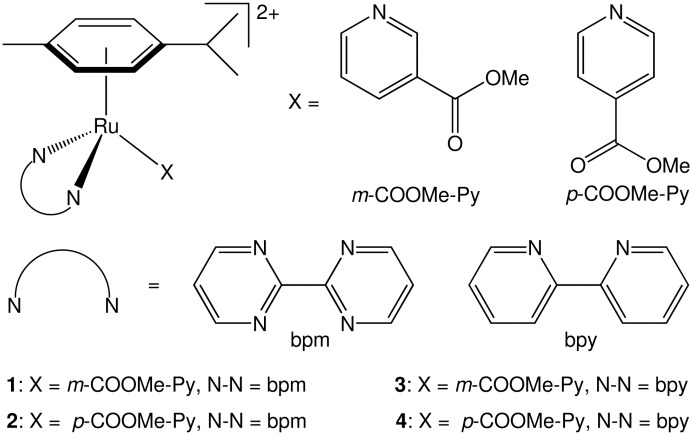
Structures of the Ru(II) arene complexes studied in this work.

In this contribution, we report on new complexes of the family [(*η*^6^-*p*-cym)Ru(N-N)(X)]^2+^ (Figure 1) (**1**–**4**) and discuss their structural, chemical and photochemical properties, including the X-ray structure for complexes **3** and **4**. The ester group on the pyridyl ligand plays a key role in conferring dark stability to all derivatives, a fundamental requirement for photoactivatable prodrugs. DFT-based computational methods were successfully used to obtain insights on the singlet and triplet excited states, allowing individuation of the dissociative state responsible for the photochemical behaviour of **1**–**4**. 

## 2. Results and Discussion

### 2.1. Synthesis and X-ray Crystal Structures

The complexes [(*η*^6^-*p*-cym)Ru(N-N)(X)]^2+^(where *p*-cym = *para*-cymene, **1**–**4**) studied in this work were synthesized as PF_6_ salts in good to moderate yields and are shown in Figure 1. Complex **2** was previously reported by some of us [13]. The synthetic route involved the reaction of the corresponding [(*η*^6^-*p*-cym)Ru(N-N)Cl][PF_6_] complex [15] with AgNO_3_ in a 1:1 mixture of MeOH/H_2_O to generate the corresponding aqua species [(*η*^6^-*p*-cym)Ru(N-N)(OH_2_)]^2+^, to which an excess of the appropriate ligand X and KPF_6_ were added. All the synthesized complexes were fully characterized by spectroscopic and analytical methods and are consistent with the structures depicted in Figure 1.

**Figure 2:**
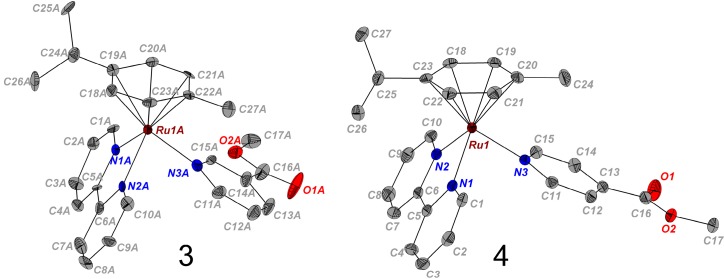
X-ray structures present in the asymmetric unit of cations [(*η*^6^-*p*-cym)Ru(bpy)(*m*-COOCH_3_-Py)]^2+^ (**3**, left) and [(*η*^6^-*p*-cym)Ru(bpy)(*p*-COOCH_3_-Py)]^2+^ (**4**, right). One of the four independent structures found in the unit cell of [(*η*^6^-*p*-cym)Ru(bpy)(*m*-COOCH_3_-Py)]^2+^ (**3**) is shown here. Thermal ellipsoids are depicted at the 50% probability level. The counter ions (PF_6_^−^) and the H atoms are omitted for clarity. The fully labelled structures and their crystal packing are depicted in the Supporting Information (Supplementary Figures S1 and S2).

The molecular structure of complexes **3** and **4** were determined by single-crystal X-ray diffraction. Crystals suitable for X-ray diffraction were obtained by slow evaporation of solutions of the complexes made up of water/methanol and acetone in the presence of excess KPF_6_ at ambient temperature. The molecular structure diagrams of the cations with numbering schemes are shown in Figure 2, the crystallographic data are listed in Table 1, while details of the crystal packing are reported in the Supporting Information (Supplementary Figures S1 and S2). Selected bond lengths and angles are given in Table 2 and Supplementary Table S1. These complexes have very similar structural features and adopt the familiar pseudo-octahedral half sandwich piano stool geometry common to other Ru(II) arene derivatives [15].

**Table 1:** Crystal data and structural refinement parameters for **3** and **4**.

	3(PF_6_)_2_	4(PF_6_)_2_
Empirical Formula	C_27_H_29_F_12_N_3_O_2_P_2_Ru	C_27_H_29_F_12_N_3_O_2_P_2_Ru
Formula weight (g·mol^−1^)	818.54	818.54
Crystal system	Monoclinic	Monoclinic
Crystal size/mm	0.52 × 0.20 × 0.10	0.50 × 0.21 × 0.16
Space group	P2(1)	P2(1)/c
Crystal	Red block	Dark-red block
a/Å	18.2866(3)	14.14530(10)
b/Å	9.55390(12)	18.6107(2)
c/Å	36.8439(5)	11.70990(10)
α/deg	90.00	90.00
β/deg	102.5101(10)	101.8020(10)
γ/deg	90.00	90.00
Volume/Å^3^	6284.10(15)	3017.51(5)
Temperature/K	100(1)	100(1)
Z	8	4
μ (CuKα) [mm^−1^]	0.704	0.733
Reflections collected	47851	20749
Independent reflections [Rint]	18934[0.032]	5932[0.028]
Parameters/restraints	2176/434	491/96
R_1_^[a]^, wR_2_^[b]^ [I > 2σ (I)]	0.0487, 0.1027	0.0255, 0.0552
R_1_^[a]^, wR_2_^[b]^ (all data)	0.0516, 0.1043	0.0295, 0.0577
GoF ^[c]^	1.108	1.108
Δρ max and min/eÅ^−3^	1.85 and −0.713	0.573 and −0.433

^[a]^ *R_1_* = Σ||Fo| − |Fc||/Σ|Fo|; ^[b]^ *wR_2_* = [Σw(Fo^2^ − Fc^2^)^2^/ΣwFo^2^)]^1/2^; ^[c]^ GoF = [Σw(Fo^2^ – Fc^2^)^2^/(*n* − *p*)]^1/2^ where *n* = number of reflections and *p* = number of parameters.

**Table 2:** Selected bond distances (Å) and angles (°) for **3** and **4**. In the case of **3** the mean values of the four independent structures present in the unit cell are reported, the data for all the structures are reported in the Supporting Information.

Bond Length (Å)/Angle (°)	3	4
Ru–arene_(centroid)_	1.708	1.702
Ru(1)–N(1)	2.084(6)	2.0851(17)
Ru(1)–N(2)	2.083(6)	2.0917(17)
Ru(1)–N(3)	2.137(7)	2.1242(17)
C(5)–C(6)	1.457(10)	1.471(3)
N(1)–Ru(1)–N(2)	77.1(3)	77.30(7)
N(1)–Ru(1)–N(3)	84.8(2)	88.16(7)
N(2)–Ru(1)–N(3)	86.6(2)	86.80(7)

The Ru atom is π-bonded to the arene ligand (*p*-cymene) and σ coordinated to the nicotinate nitrogen and to the two nitrogen atoms of the chelating ligand (bpy) [15]. Nevertheless, there some significant differences in the Ru nitrogen bond lengths between the chelating N atoms (2.084(6) Å and 2.083(6) Å for **3**, and 2.0917(17) Å and 2.0851(17) Å for **4**) and the Ru–N bond length of the mono-coordinated N (2.137(7) Å, 2.1242(17) Å) of **3** and **4**, respectively. The longer bond length in the latter case inferring that it is relatively labile. This was also found to be the case in the analogous Ru(II) arene complexes containing N-N chelated ligands [13].

Moreover, there are some structural differences between **3** and **4**. The presence the methyl ester group in the meta position (complex **3**) imposes some steric constraints around the central Ru atom, resulting in the narrowing of the of the N2A–Ru–N3A bond angle (84.8°) for complex **3**, compared to N1–Ru–N3 (88.16°) for complex **4**. The position of the methyl ester group of the N-σ-donor ligand has no influence on the corresponding Ru(II)–arene_(centroid)_ distances (~1.70 Å) and is consistent with the values reported for similar complexes [13].

Analogues of **1**–**4** bearing *m/p*-carboxypyridine (*i.e*., nicotinic and isonicotinic acid) were found to be unstable in DMSO and aqueous solutions in the dark. Dissolution of these compounds in the two solvents at 310 K readily gave rise to ligand exchange reactions as indicated by the concomitant changes in appearance of a new set of peaks in the ^1^H-NMR spectra, which correspond to the aqua adduct and to signals for either the free nicotinic or isonicotinic acids, as applicable. For example, the extent of the hydrolysis for the isonicotic analogue of **4** was already *ca.* 40%–50% after <15 min as observed by ^1^H-NMR (*data not shown*). However, the complexes containing the esterified analogues *i.e*., **1**–**4** were found to be stable towards hydrolysis for over 48 hours under the same experimental conditions and were selected for further studies into ligand exchange as a function of photoactivation (*vide infra*).

### 2.2. DFT Geometries and Electronic Structures of **1**–**4**

Geometry optimization of complexes **1**–**4** was performed for both the ground state and the lowest-lying triplet state. Details of the main optimized geometrical parameters are summarized in Table 3 and in Supplementary Tables S1–S3.

**Table 3:** Selected calculated bond distances (Å) for **1**–**4** in the ground state (S0) and lowest-lying triplet state (T0 and T1) geometries.

Compound	Ru–N(L)	Ru–N(N-N)	Ru–N(N-N)	Ru–arene_(centroid)_
**S0**				
**1**	2.157	2.111	2.109	1.845
**2**	2.147	2.113	2.111	1.850
**3**	2.160	2.102	2.098	1.848
**4**	2.148	2.104	2.102	1.853
**T0**				
**1**	2.140	2.439	2.130	2.083
**2**	2.136	2.454	2.137	2.094
**3**	2.152	2.386	2.112	2.092
**4**	2.153	2.391	2.110	2.096
**T1**				
**1**	2.556	2.103	2.099	2.123
**2**	2.522	2.105	2.093	2.137
**3**	2.565	2.084	2.087	2.142
**4**	2.532	2.088	2.082	2.153

In the ground state (S0), all the complexes have a pseudo-octahedral coordination structure. Bond lengths and angles obtained by DFT calculations are in excellent agreement with the experimental X-ray diffraction data collected for complexes **3** and **4** (Table 3).

The lowest-lying triplet state geometries were also optimized, due to the key role that this state can play in the photochemistry of Ru(II) arene complexes. The geometry optimization of **1**–**4** in the triplet electronic state led to two distinct triplets, T0 and T1. The former is the actual lowest-lying triplet (T0) while the latter (T1) has a slightly higher energy (about 0.1 eV). Frequency calculations confirmed that both these geometries correspond to a minimum in the potential energy surface.

In both T0 and T1 states, **1**–**4** display a distorted geometry. In T0, complexes have similar Ru–N(methyl nicotinate) distances of 2.145 ± 0.009 Å, which resemble those observed in the ground state S0 (2.153 ± 0.006 Å), while one of the Ru–N(N-N) bond distances is considerably longer than the other, typically ~2.12 and 2.40 Å. In contrast, in the T1 state **1**–**4** show considerably elongated Ru–N(methyl nicotinate) distances (~2.54 Å) and the Ru–N(N-N) distances resemble that obtained for the ground state. In both T0 and T1 states, each of the computed Ru–*η*^6^-*p*-cymene(centroid) distances are longer than those calculated for the ground state (~2.1 Å).

**Figure 3:**
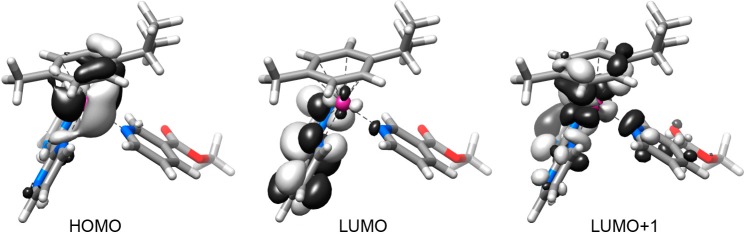
Selected frontier orbitals for complex **1** in the ground state (S0) geometry.

Complexes **1**–**4** display similar frontier molecular orbitals in the S0 state (Figure 3 for **1** and Supplementary Figure S2). They all have the HOMO localized on the metal atom and on the *η*^6^-*p*-cymene ligand while the LUMO is centred on the 2,2'-bipyrimidine (**1**, **2**) or on the 2,2'-bipyridine unit (**3**, **4**). The following four virtual orbitals (from LUMO+1 to LUMO+4) display a σ-antibonding feature towards the Ru–N(methyl nicotinate) bond or one of the Ru–N(N-N) bonds.

**Figure 4:**
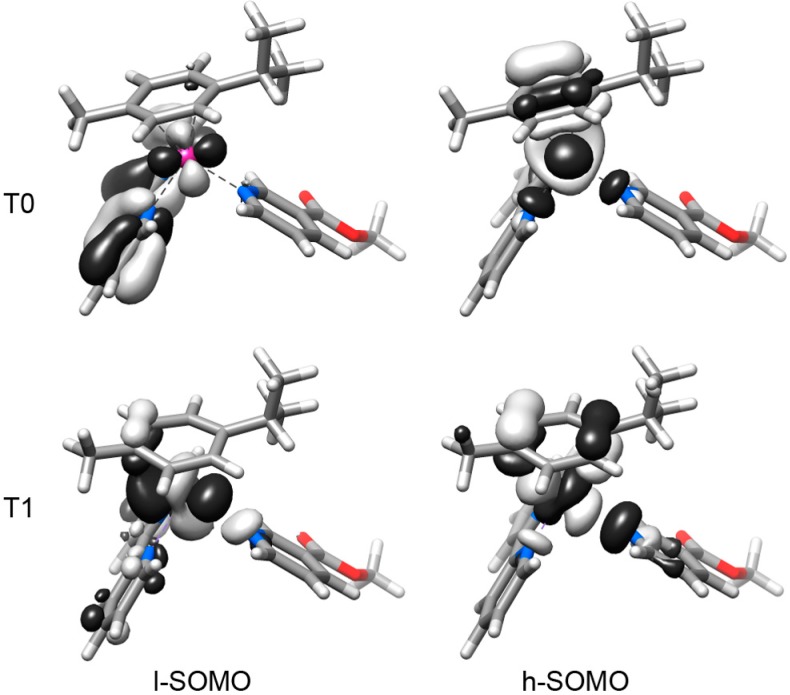
Calculated lowest- and highest-Single Occupied Molecular Orbitals (h-SOMO and l-SOMO), for complex **3** in the T0 and T1 optimized geometries.

In the triplet electronic state (Figure 4 for **3** and Supplementary Figures S4 and S5), the complexes display a lowest-SOMO (l-SOMO) delocalized over the whole molecule, both at the lowest-lying triplet (T0) optimized geometry and at the T1 optimized geometry. Only for **3** and **4** at the T0 geometry the l-SOMO is mainly localized on the N-N chelating ligand. The T0 highest-SOMO (h-SOMO) is delocalized over the *η*^6^-*p*-cymene ligand, the Ru atom and the N-N chelating ligand of **1**–**4** in the lowest-lying triplet state. On the contrary, when **1**–**4** are in the T1 state, their h-SOMO is delocalized over the *η*^6^-*p*-cymene ligand, the Ru atom and the methyl nicotinate ligand. In both T0 and T1, the h-SOMO of **1**–**4** has a dissociative nature, due to the σ-antibonding character of this orbital towards one of the Ru–N(N-N) bonds (T0 state) or the Ru–N(methyl nicotinate) bond (T1 state).

### 2.3. Photophysical and Photochemical Properties of **1**–**4**

**Figure 5:**
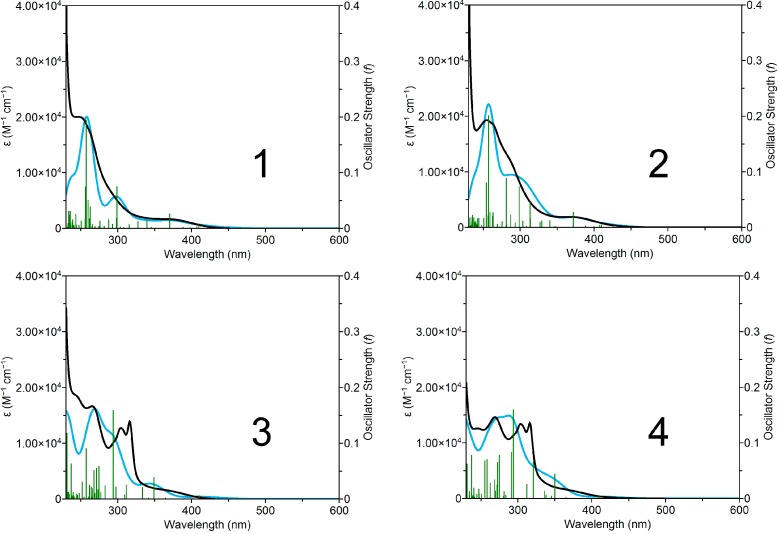
Experimental (black) and theoretical (light blue) UV-Vis spectra of **1**–**4** in aqueous solution. Calculated singlet-singlet electronic transitions are shown as vertical bars with heights equal to their oscillator strength. GaussSum 2.2.5 [10] was used to simulate the theoretical UV-Vis spectra (FWHM = 3000 cm^−1^).

The experimental and calculated UV–vis absorption spectra for **1**–**4** are reported in Figure 5. A complete set of TD-DFT calculations was performed at the B3LYP/LanL2Dz/6-31G** level to characterize their singlet excited states and electronic properties.

According to DFT, the intense absorption bands of **1**–**4** in the high energy range (*ca.* 340–250 nm) are described by a series of metal-to-ligand charge transfer (^1^MLCT) transitions alternated with few intense transitions having a pronounced interligand (^1^IL) or ligand-centered (^1^LC) character.

The absorbance tail in the 380–450 nm region is dominated by metal-to-ligand charge transfer (^1^MLCT) transitions. Some of these transitions are partially dissociative, since they have significant contributions from the Ru–N(N-N) and Ru–N(L) σ-antibonding orbitals.

**Figure 6:**
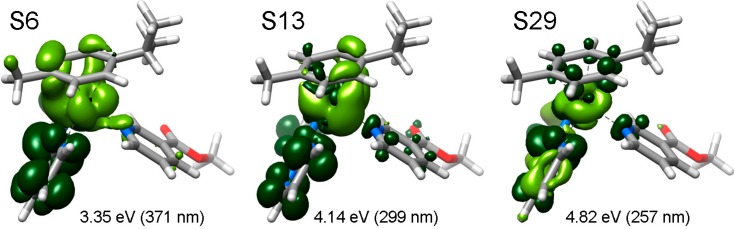
Selected electron density difference maps (EDDMs) of singlet excited state transitions of **1** in water (B3LYP/LANL2DZ/6-31G** level, PCM method). Light green indicates a decrease in electron density, while green indicates an increase.

Electron density difference maps (EDDMs) were used to visualize the character associated with the given transition. EDDMs are obtained subtracting the initial electron density of **1**–**4** (*i.e*., before the electronic transition) to their electron density in the excited state (*i.e*., after the electronic transition). Selected EDDMs for complex **1** are shown in Figure 6 as an example and in the Supporting Information (Supplementary Figures S6–S9) section for a full account. The orbital compositions of computed singlet transitions for all complexes are reported in Supplementary Tables S5–S8.

**Figure 7:**
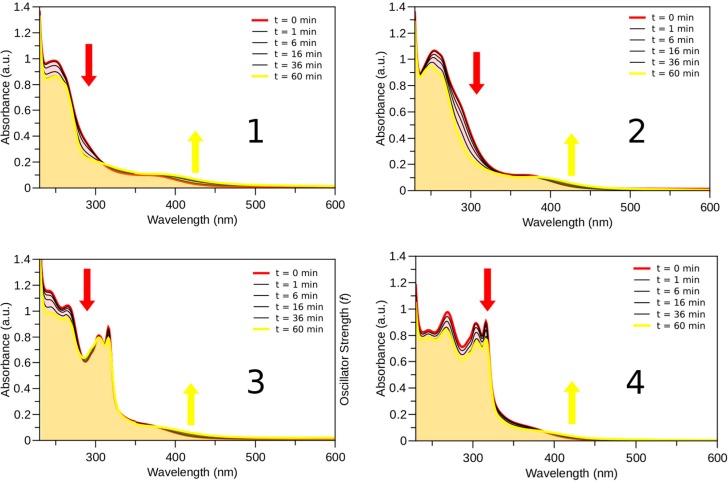
Photolysis (λ_exc_ = 400 nm, 2 mW·cm^−2^) of complexes **1**–**4** (60 μM) in aqueous solution followed by UV-Vis.

**Figure 8:**
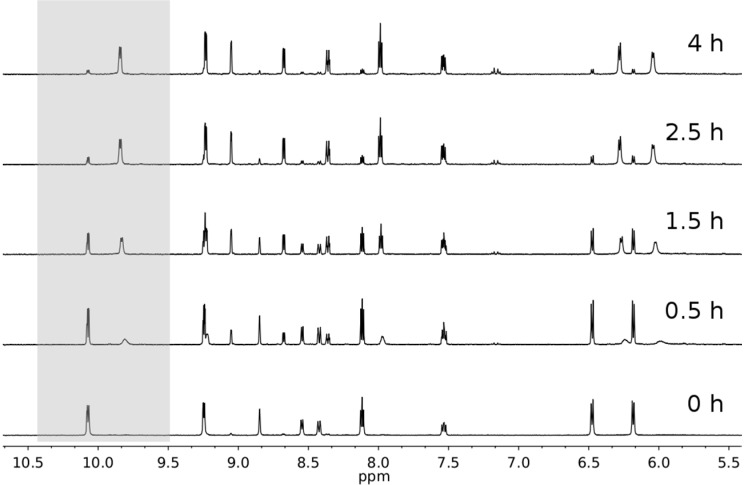
Photolysis (λ_exc_ = 400 nm, 2 mW·cm^−2^) of complex **1** (*ca.* 300 μM) in aqueous solution (1%–5% DMSO) followed by ^1^H-NMR spectroscopy. The grey panel highlights the disappearance of the bpm signal relative to the proton in position 6,6' for **1** and the appearance of the analogue signal for its aqua derivative [(*η*^6^-*p*-cym)Ru(bpm)H_2_O]^2+^.

In aqueous solution both sets of complexes behave similarly upon light irradiation (λ_exc_ = 400 nm). As observed previously [13,14], the monodentate pyridyl ligands are released and formation of the aqua complexes [(*η*^6^-*p*-cym)Ru(N-N)(OH_2_)]^2+^ is observed. The reaction progression is easily monitored by UV-Vis following the decrease in intensity of the bands in the UV spectrum and the increase of the absorption at λ > 400 nm (Figure 7). The presence of pseudoisosbestic points indicates the photosubstitution reaction is clean, giving only a single photoproduct, as confirmed by the NMR photolysis experiment reported for complex **1** (Figure 8). Indeed, new sets of NMR signals are formed upon light excitation, corresponding to [(*η*^6^-*p*-cym)Ru(N-N)(OH_2_]^2+^ complexes and free *m*-COOCH_3_-Py. Estimation of the photosubstitution yields with actinometry indicated that complexes **1**–**4** are modestly photoreactive, since they display yields smaller than 1% (Supplementary Table S9).

As shown by the TD-DFT calculations, the selective photochemical dissociation of the methyl nicotinate ligand is consistent with the presence of σ-antibonding orbitals that participate in several singlet transitions. In particular, the antibonding orbitals L+1 and L+3 participate in all the transitions between 300 and 440 nm of **1**. Similarly, the antibonding orbital L+4 is involved in all the transitions between 330 and 440 nm of **2**. In the case of **3**, only few transitions involve the antibonding orbital L+3. Finally, in the case of the less photoactive complex (**4**), there are no contributions from the antibonding orbital L+1 to low energy transitions (over 330 nm).

On the basis of the DFT results, as well as modest photochemistry rates [13,14], triplet states are believed to play a key role in the ligand photodissociation mechanism of these Ru(II) arene complexes. The distorted geometry, exhibited by **1**–**4** in their T0 and T1 states, can be ascribed to the population of σ-antibonding h-SOMOs, involving one Ru–N bond. In the lowest-lying triplet state T0, the dissociation of the chelating ligand is prevented by the strong coordination of the other ring to the Ru atom, which makes this state less likely to prompt release of ligands [16,17]. On the contrary, the elongation of the Ru–N(methyl nicotinate) bond of **1**–**4** in the T1 state can be related to the dissociation of the methyl nicotinate units. Spin density surfaces (Supplementary Figure S10) show that the nature of the dissociative (T1) and nondissociative (T0) states of all the complexes is ^3^MC. The nondissociative ^3^MC states (T0) are responsible for the return of the excited molecules to the ground state, while the dissociative ^3^MC states cause the selective dissociation of the methyl nicotinate ligand.

## 3. Experimental Section

### 3.1. General Information

RuCl_3_·3H_2_O was purchased from Precious Metals Online (PMO Pty Ltd., Wollongong, Australia) and used as received. 2,2'-bipyrimidine (bpm), 2,2'-bipyridine (bpy) silver nitrate (AgNO_3_), potassium hexafluorophosphate (KPF_6_), isonicotinic acid, nicotinic acid, methyl nicotinate, methyl isonicotinate were obtained from Sigma-Aldrich (St. Louis, MO, USA). The dimer [(*η*^6^-*p*-cym)RuCl_2_]_2_ was prepared based on literature methods [18,19]. The Ru(II) arene complexes [(*η*^6^-*p*-cym)Ru(bpm)Cl][PF_6_] and [(*η*^6^-*p*-cym)Ru(bpy)Cl][PF_6_] were synthesised following a method previously described [15]. UV-Vis absorption spectra were recorded on a Cary 5000 spectrophotometer (Agilent Technologies, Santa Clara, CA, USA) using 1-cm path length quartz cuvettes (1 mL) and a PTP1 Peltier temperature controller. Spectra were recorded at 310 K in deionized water from 200 to 800 nm. ^1^H and ^13^C-NMR spectra were acquired in 5 mm NMR tubes at 298 K (unless otherwise stated) on a Bruker Avance 500 NMR spectrometer (Bruker Corporation, Billerica, MA, USA). ^1^H chemical shifts were internally referenced to TMS via residual DMSO (δ 2.50). 1D spectra were recorded using standard pulse sequences. Photolysis studies were performed by irradiating aqueous solutions (1%–5% DMSO) of complexes **1**–**4** with a Xe lamp (KiloArc) at 400 nm (2 mW·cm^−2^). Formation of photoproducts was monitored by UV-vis spectroscopy (60 μM) and by NMR (300 μM). The quantum yields (Φ) for photoinduced ligand substitution of **1**–**4** were determined by ferrioxalate actinometry [20] under 395-nm light irradiation, using a MWLLS-11 Fiber Coupled 11 LED Multi-Wavelength LED Light Source (15.1 mW·cm^−2^) by Prizmatix Ltd (Givat-Shmuel, Israel). Details are reported in the Supporting Information. Elemental analyses were performed by the Warwick Analytical Service, which is the analytical division of Exeter Analytical Ltd, using a CE440 Elemental Analyzer (Exeter Analytical Ltd, Coventry, UK).

### 3.2. Synthesis

The complexes **1**–**4** were synthesised using a similar procedure previously reported [21]. An aluminium-foil-covered round bottom flask was charged with [(*η*^6^-*p*-cym)Ru(bpy)Cl][PF_6_] and a 1 mol equivalent of AgNO_3_ in a 1:1 mixture of MeOH/H_2_O (10 mL) and the reaction mixture stirred at 323 K (4 h). Precipitated AgCl was then removed by filtration. To the clear filtrate up to 10 mol equivalents of the appropriate pyridine derivative was added and the reaction mixture left stirring overnight at ambient temperature. The volume of the reaction mixture was reduced by rotary evaporation and up to 5 mol equivalents of KPF_6_ was added. The precipitate that formed was filtered and redissolved by the addition of acetone and the clear reaction mixture was left to evaporate slowly at ambient temperature. The crystalline materials that were formed were collected by filtration, washed with methanol and ether and dried in air. Quantitative details of the preparations and the nature of the product of the individual reactions are described below.

#### 3.2.1. [(*η*^6^-*p*-cym)Ru(bpm)(*m*-COOMe-Py)]^2+^ (**1**)

The complex was prepared as described above: AgNO_3_ (0.029 g, 0.174 mmol), MeOH/H_2_O (1:1, 20 mL), [(*η*^6^-*p*-cym)Ru(bpm)Cl][PF_6_] (0.10 g, 0.174 mmol). To the clear solution of [(*η*^6^-*p*-cym)Ru(bpm)H_2_O]^2+^, methyl-nicotinate (0.120 g, 0.87 mmol) and KPF_6_ (0.220 g, 1.2 mmol) was added. Red crystals were obtained. Yield: (72 mg, 40%). ^1^H-NMR (D_2_O/DMSO-d_6_ (5%), ppm) δ: 10.02 (dd, bpm, ^3^*J*_HH_ = 5.8 and 2.0 Hz, 2H), 9.24 (dd, bpm, ^3^*J*_HH_ = 4.8 and 1.9 Hz, 2H), 8.59 (d, R-Py, ^3^*J*_HH_ = 6.1 Hz, 2H), 8.11 (dd, bpm, ^3^*J*_HH_ = 5.9 and 4.8 Hz, 2H), 7.85 (d, R-Py, ^3^*J*_HH_ = 6.1 Hz, 2H), 6.47 (d, p-cym, ^3^*J*_HH_ = 6.5 Hz, 2H), 6.16 (d, p-cym, ^3^*J*_HH_ = 6.5 Hz, 2H), 3.85 (s, COO*CH_3_*, 3H), 2.42 (hept, p-cym *CH*, ^3^*J*_HH_ = 7.0 Hz, 1H), 1.79 (s, p-cym *CH_3_*, 3H), 0.88 (d, p-cym *CH_3_*, ^3^*J*_HH_ = 6.9 Hz, 6H). ^13^C-NMR (DMSO-d_6_, ppm) δ: 164.2, 164.1, 161.5, 160.7, 155.1, 140.0, 126.1, 125.8, 107.6, 106.7, 90.8, 86.2, 53.8, 30.4, 22.2, 17.8. Elemental Analysis: Calculated for C_26_H_29_F_12_N_3_O_2_P_2_Ru.0.5(CH_3_)_2_CO: H 3.73, C 39.52, N 5.03; found H 3.35, C 39.48, N 4.99.

#### 3.2.2. [(*η*^6^-*p*-cym)Ru(bpm)(*p*-COOMe-Py)]^2+^ (**2**)

The complex was prepared as described above: AgNO_3_ (0.029 g, 0.174 mmol), MeOH/H_2_O (1:1, 20 mL), [(*η*^6^-*p*-cym)Ru(bpm)Cl][PF_6_] (0.10 g, 0.174 mmol). To the clear solution of [(*η*^6^-*p*-cym)Ru(bpm)H_2_O]^2+^, methyl-isonicotinate (0.120 g, 0.87 mmol) and KPF_6_ (0.220 g, 1.2 mmol) was then added. Reddish yellow crystals were obtained. Yield: (42 mg, 29%). ^1^H-NMR (D_2_O/DMSO-d_6_ (5%), ppm) δ: 10.07 (dd, bpm, ^3^*J*_HH_ = 5.8 and 1.9 Hz, 2H), 9.25 (dd, bpm, ^3^*J*_HH_ = 4.8 and 1.9 Hz, 2H), 8.85 (s, R-Py, 1H), 8.55 (d, R-Py, ^3^*J*_HH_ = 5.7 Hz, 1H), 8.42 (d, R-Py, ^3^*J*_HH_ = 8.0 Hz, 1H), 8.13 (dd, bpm, ^3^*J*_HH_ = 5.8 and 4.8 Hz, 2H), 7.53 (t, R-Py, ^3^*J*_HH_ = 8.1 and 5.8 Hz, 1H), 6.48 (d, p-cym, ^3^*J*_HH_ = 6.5 Hz, 2H), 6.18 (d, p-cym, ^3^*J*_HH_ = 6.5 Hz, 2H), 3.87 (s, COO*CH_3_*, 3H), 2.43 (hept, p-cym *CH*, ^3^*J*_HH_ = 7.0 Hz, 1H), 1.80 (s, p-cym *CH_3_*, 3H), 0.88 (d, p-cym *CH_3_*, ^3^*J*_HH_ = 6.8 Hz, 6H).^13^C-NMR (DMSO-d_6_, ppm) δ: 164.0, 161.6, 160.9, 157.3, 153.9, 140.5, 128.9, 127.4, 126.0, 107.5, 106.5, 90.6, 86.2, 56.5, 53.5, 30.4, 22.2, 19.0, 17.8. Elemental Analysis: Calculated for C_26_H_29_F_12_N_3_O_2_P_2_Ru. 0.5(CH_3_)_2_CO: H 3.73, C 39.52, N 5.03; found H 3.34, C 39.44, N 5.01.

#### 3.2.3. [(*η*^6^-*p*-cym)Ru(bpy)(*m*-COOMe-Py)]^2+^ (**3**)

The complex was prepared as described above: AgNO_3_ (0.04 g, 0.235 mmol), MeOH/H_2_O (1:1, 20 mL), [(*η*^6^-*p*-cym)Ru(bpy)Cl][PF_6_] (0.10 g, 0.175 mmol). To the clear solution of [(*η*^6^-*p*-cym)Ru(bpy)H_2_O]^2+^, methyl-nicotinate (0.165 g, 1.2 mmol) and KPF_6_ (0.220 g, 1.2 mmol) was then added. The yellow needles obtained were collected by filtration and washed with methanol and ether and dried in air in the dark. Some of the crystals were used for single crystal X-ray analysis. Yield: (72 mg, 50%). ^1^H-NMR (D_2_O/DMSO-d_6_ (5%), ppm) δ: 9.70 (d, bpy, ^3^*J*_HH_ = 7.4 Hz, 2H), 8.80 (s, R-Py, 1H), 8.55 (d, R-Py, ^3^*J*_HH_ = 5.5 Hz, 1H), 8.37 (dt, R-Py, ^3^*J*_HH_ = 8.1 and 1.7 Hz, 1H), 8.30 (d, bpy, ^3^*J*_HH_ = 8.0 Hz, 2H), 8.23 (td, bpy, ^3^*J*_HH_ = 8.0 and 1.5 Hz, 2H), 7.90 (td, bpy, ^3^J_HH_ = 7.4 and 1.5 Hz, 2H), 7.47 (dd, R-Py, ^3^*J*_HH_ = 8.1 and 5.8 Hz, 1H), 6.44 (d, p-cym, ^3^*J*_HH_ = 6.4 Hz, 2H), 6.05 (d, p-cym, ^3^*J*_HH_ = 6.4 Hz, 2H), 3.85 (s, COO*CH_3_*, 3H), 2.42 (hept, p-cym *CH*, ^3^*J*_HH_ = 7.0 Hz, 1H), 1.77 (s, p-cym *CH_3_*, 3H), 0.83 (d, p-cym *CH_3_*, ^3^*J*_HH_ = 6.9 Hz, 6H). ^13^C-NMR (DMSO-d_6_, ppm) δ: 163.5, 156.9, 156.6, 155.4, 152.8, 141.8, 140.7, 129.5, 128.9, 127.5, 125.2, 108.6, 103.8, 92.3, 85.1, 53.5, 30.6, 22.2, 17.9. Elemental Analysis: Calculated for C_24_H_27_F_12_N5O_2_P_2_Ru. 0.5(CH_3_)_2_CO: H 3.60, C 36.56, N 8.36; found H 3.29, C 36.32, N 8.16.

#### 3.2.4. [(*η*^6^-*p*-cym)Ru(bpy)(*p*-COOMe-Py)]^2+^ (**4**)

The complex was prepared in an identical manner as described for **3**. Red crystals were collected by filtration and washed with methanol and ether and dried in air in the dark. Some of the crystals were used for single crystal X-ray analysis. Yield: (50 mg, 35%). ^1^H-NMR (D_2_O/DMSO-d_6_ (5%), ppm) δ: 9.66 (d, bpy, ^3^*J*_HH_ = 5.9 Hz, 2H), 8.57 (d, R-Py, ^3^*J*_HH_ = 7.0 Hz, 2H), 8.30 (d, bpy, ^3^*J*_HH_ = 8.1 Hz, 2H), 8.23 (t, bpy, ^3^*J*_HH_ = 7.7 Hz, 2H), 7.88 (t, bpy, ^3^*J*_HH_ = 6.0 Hz, 2H), 7.80 (d, R-Py, ^3^*J*_HH_ = 7.0 Hz, 2H), 6.43 (d, p-cym, ^3^*J*_HH_ = 6.4 Hz, 2H), 6.02 (d, p-cym, ^3^*J*_HH_ = 6.4 Hz, 2H), 3.84 (s, COO*CH_3_*, 3H), 2.41 (hept, p-cym *CH*, ^3^*J*_HH_ = 7.0 Hz, 1H), 1.75 (s, p-cym *CH_3_*, 3H), 0.83 (d, p-cym *CH_3_*, ^3^*J*_HH_ = 6.9 Hz, 6H). ^13^C-NMR (DMSO-d_6_, ppm) δ: 164.0, 156.7, 155.3, 154.4, 141.7, 140.3, 129.6, 125.9, 125.1, 108.7, 104.1, 92.5, 85.1, 53.7, 30.6, 22.2, 17.9. Elemental Analysis: Calculated for C_24_H_27_F_12_N_5_O_2_P_2_Ru. 0.5(CH_3_)_2_CO: H 3.60, C 36.56, N 8.36; found H 3.27, C 36.56, N 8.42.

### 3.3. X-ray Crystallography

Diffraction data were collected on Agilent Super Nova Mo-diffractometer (Agilent Technologies, Santa Clara, CA, USA), equipped with CCD area detector, at a temperature of 100 K, equipped with an Agilent 700 Cryosystem Cryostream Cooler fed with liquid nitrogen. All structures were refined by full-matrix least squares against *F*^2^ using SHELXL-97 [22] and were solved by direct methods [23,24,25]. Hydrogen atoms were added at calculated positions and refined using a riding model. X-ray crystallographic data for complexes **3** and **4** have been deposited in the Cambridge Crystallographic Data Centre under the accession numbers CCDC 1018418 and 1018419, respectively. 

### 3.4. Computational Details

All calculations were performed with the Gaussian 09 (G09) program package [26], employing the DFT and TD-DFT methods [27,28], the Becke three-parameter hybrid functional [29], and the Lee-Yang-Parr’s gradient corrected correlation functional (B3LYP) [30]. The solvent effect was included using the polarizable continuum model (PCM method) [31,32], with water as solvent. The LanL2DZ basis set [33] and effective core potential were used for the Ru atom and the 6-31G** basis set [34] was used for all the other atoms. The B3LYP/LanL2DZ/6-31G** combination was selected since it previously provided satisfactory results on similar ruthenium arene complexes [13,14].

Geometry optimizations (ground state (S0), lowest-lying triplet state (T0 and T1)) were carried out without any symmetry constraints, the nature of all stationary points was confirmed by normal-mode analysis and no imaginary frequencies were found.

The UV-Vis electronic absorption spectra were simulated by TD-DFT [27,28], computing a total of 50 singlet excited states. Eight triplet excited states were calculated by TD-DFT using the lowest-lying triplet state geometry. The electronic distribution and the localization of the singlet and triplet excited states were visualized using electron density difference maps (EDDMs).

GaussSum 2.2.5 [35] was used to simulate the theoretical UV-Vis spectra and for EDDMs calculations [36,37]. Molecular graphics images were produced using the UCSF Chimera package from the Resource for Biocomputing, Visualization, and Informatics at the University of California, San Francisco (supported by NIH P41 RR001081) [38]. A full summary of the computational results is reported in the Supporting Information.

## 4. Conclusions

Several new Ru(II) arene complexes of formula [(*η*^6^-*p*-cym)Ru(N-N)(X)]^2+^(where *p*-cym = *para*-cymene, N-N = bpm or bpy and X = *m/o*-substituted pyridine) have been synthesised and characterized in this work, including the X-ray structure of two of them, namely [(*η*^6^-*p*-cym)Ru(bpy)(*m*-COOCH_3_-Py)]^2+^ (**3**) and[(*η*^6^-*p*-cym)Ru(bpy)(*p*-COOCH_3_-Py)]^2+^ (**4**). Pyridyl ester derivatives (**1**–**4**) are stable in aqueous solution and do not hydrolyze in the dark within the monitored period (48 h). On the contrary, their carboxylic analogues are not stable in the dark and readily dissociate the pyridyl ligand affording the [(*η*^6^-*p*-cym)Ru(N-N)(OH_2_)]^2+^ complex. This is a crucial discovery for the design of novel photoactivatable Ru(II) arene complexes which are required to be substitutionally inert in the dark since there are evidences that aqua species play a fundamental role in their cytotoxicity mechanism [39]. Moreover, such result highlights how a subtle change in the electronic structure of the periphery of the monodentate pyridyl ligand (COOMe *vs* COOH) can affect the overall stability of the complex.

Notably, pyridyl esters could also offer new synthetic routes for functionalization of Ru(II) arene complexes with dyes [40] or targeting/anchoring groups suitable for coupling to proteins or decoration of nanoparticles, strategies that could be used to favour the cellular delivery of this class of agents.

Finally, DFT calculations provided a valuable rationalization of the photochemistry of complexes **1**–**4**, pinpointing for the first time that the dissociative ll-T1 triplet state is likely to be responsible for the selective light-induced ejection of the monodentate pyridyl ligand.

## Supplementary Materials

Supplementary materials can be accessed at: http://www.mdpi.com/1420-3049/20/04/7276/s1.
