# Supplementary material for: Photorelease of Pyridyl Esters in Organometallic Ru(II) Arene Complexes
Source: Molecules. 2015 Apr 21;20(4):7276–91. doi: 10.3390/molecules20047276 (PMC6272714; doi:10.3390/molecules20047276)
Supplement: Supplementary file 1 [file molecules-20-07276-s001.pdf]

Supporting Information

Table S1. Selected bond distances (Å) and angles (°) for the four independent structures present in the unit cell of **3**.

Bond Length (Å)/Angle (°)	3a	3b	3c	3d	Mean Values
Ru–arene$_{(centroid)}$	1.697	1.702	1.716	1.715	1.708
Ru(1D)–N(1D)	2.085(5)	2.098(6)	2.067(6)	2.084(7)	2.084
Ru(1D)–N(2D)	2.083(5)	2.089(6)	2.080(6)	2.078(6)	2.083
Ru(1D)–N(3D)	2.118(6)	2.147(6)	2.126(5)	2.155(11)	2.137
C(5)–C(6)	1.479(8)	1.454(12)	1.475(9)	1.421(9)	1.457
N(1)–Ru(1)–N(2)	76.8(2)	77.6(3)	76.9(2)	77.0(3)	77.1
N(1)–Ru(1)–N(3)	83.8(2)	84.8(2)	84.6(2)	85.9(4)	84.8
N(2)–Ru(1)–N(3)	86.3(2)	86.7(2)	87.9(2)	85.7(4)	86.6

Table S2. Selected calculated bond distances (Å) for **1**–**4** in the ground state (S0) geometry.

Compound	Ru–N(L)	Ru–N(N,N')	Ru–N(N,N')	Ru–arene$_{(centroid)}$
**1**	2.157	2.111	2.109	1.845
**2**	2.147	2.113	2.111	1.850
**3**	2.160	2.102	2.098	1.848
**4**	2.148	2.104	2.102	1.853

Table S3. Selected calculated bond distances (Å) for **1**–**4** in the lowest-lying triplet state (T0) geometry.

Compound	Ru–N(L)	Ru–N(N,N')	Ru–N(N,N')	Ru–arene$_{(centroid)}$
**1**	2.140	2.439	2.130	2.083
**2**	2.136	2.454	2.137	2.094
**3**	2.152	2.386	2.112	2.092
**4**	2.153	2.391	2.110	2.096

Table S4. Selected calculated bond distances (Å) for **1**–**4** in the T1 triplet state geometry.

Compound	Ru–N(L)	Ru–N(N,N')	Ru–N(N,N')	Ru–arene$_{(centroid)}$
**1**	2.556	2.103	2.099	2.123
**2**	2.522	2.105	2.093	2.137
**3**	2.565	2.084	2.087	2.142
**4**	2.532	2.088	2.082	2.153

Table S5. Selected TDDFT singlet transitions for complex **1**.

Transition Number	Energy (eV)	Wavelength (nm)	Oscillator Strength	Major Contributions
1	2.81	441	0.0021	HOMO→LUMO (10%), HOMO→L+1 (30%), HOMO→L+2 (11%), HOMO→L+3 (21%)
2	3.03	410	0.0060	H−2→L+3 (15%), H−1→L+3 (19%), HOMO→L+2 (10%)
3	3.04	407	0.0023	H−1→L+1 (19%), H−1→L+3 (23%), HOMO→L+2 (13%), HOMO→L+5 (12%)
4	3.12	398	0.0008	H−2→L+1 (11%), H−2→L+2 (19%), H−2→L+5 (15%), H−1→L+2 (10%), HOMO→LUMO (17%)
5	3.19	388	0.0017	HOMO→LUMO (65%), HOMO→L+3 (11%)
6	3.35	371	0.0259	H−1→LUMO (78%)
8	3.65	339	0.0147	H−2→LUMO (41%), H−2→L+3 (15%)
9	3.79	327	0.0124	H−2→LUMO (47%), H−2→L+3 (25%)
13	4.14	299	0.0748	H−1→L+1 (10%), HOMO→L+2 (10%), HOMO→L+3 (11%), HOMO→L+5 (42%)
14	4.16	299	0.0180	HOMO→L+4 (84%)
16	4.31	288	0.0154	H−1→L+4 (91%)
19	4.49	276	0.0131	H−3→LUMO (10%), H−2→L+1 (37%), H−2→L+2 (35%)
26	4.72	263	0.0383	H−6→LUMO (14%), H−5→L+1 (19%), H−5→L+2 (17%)
27	4.73	262	0.0170	H−2→L+4 (24%), H−1→L+7 (22%), HOMO→L+8 (34%)
28	4.77	260	0.0504	H−2→L+4 (33%), H−1→L+6 (19%), H−1→L+8 (12%), HOMO→L+8 (14%)
29	4.82	257	0.1939	H−6→LUMO (38%)
30	4.84	256	0.0741	H−5→L+5 (10%), H−1→L+7 (14%), H−1→L+8 (17%)
31	4.84	256	0.0160	H−5→L+5 (11%), H−1→L+7 (11%), H−1→L+8 (20%)
32	4.94	251	0.0132	H−9→LUMO (61%), H−7→LUMO (22%)

Table S6. Selected TDDFT singlet transitions for complex **2**.

Transition Number	Energy (eV)	Wavelength (nm)	Oscillator Strength	Major Contributions
1	2.81	441	0.0018	HOMO→L+2 (33%), HOMO→L+4 (24%)
2	3.03	410	0.0042	H−2→L+4 (15%), H−1→L+4 (12%), HOMO→L+2 (13%), HOMO→L+4 (11%), HOMO→L+5 (12%)
3	3.04	408	0.0037	H−1→LUMO (11%), H−1→L+2 (18%), H−1→L+4 (38%)
4	3.10	400	0.0009	H−2→L+2 (22%), H−2→L+3 (12%), H−2→L+5 (14%), H−1→L+2 (12%), HOMO→LUMO (13%)
5	3.19	388	0.0021	HOMO→LUMO (70%), HOMO→L+4 (11%)
6	3.33	372	0.0273	H−1→LUMO (79%)
8	3.64	340	0.0117	H−2→LUMO (40%), H−2→L+4 (18%)
9	3.76	329	0.0121	H−2→LUMO (20%), H−2→L+4 (10%), HOMO→L+1 (54%)

Table S6. *Cont.*

Transition Number	Energy (eV)	Wavelength (nm)	Oscillator Strength	Major Contributions
11	3.95	314	0.0159	H−1→L+1 (12%), HOMO→L+2 (25%), HOMO→L+3 (49%)
12	3.96	313	0.0463	H−1→L+1 (68%), HOMO→L+3 (11%)
14	4.09	303	0.0111	H−1→L+2 (24%), H−1→L+3 (59%)
15	4.15	299	0.0727	HOMO→L+5 (58%)
17	4.32	287	0.0220	H−3→L+1 (72%), H−2→L+1 (16%)
25	4.71	263	0.0262	H−6→LUMO (13%), H−5→L+3 (22%), HOMO→L+8 (13%)
26	4.72	262	0.0196	H−1→L+6 (15%), H−1→L+7 (14%), HOMO→L+8 (32%)
27	4.79	259	0.0268	H−1→L+6 (19%), H−1→L+8 (46%)
28	4.82	257	0.2008	H−6→LUMO (38%)
29	4.83	256	0.0208	H−7→L+3 (15%), H−5→L+5 (22%)
30	4.88	254	0.0804	H−4→L+1 (64%)
31	4.94	251	0.0162	H−9→LUMO (62%), H−7→LUMO (21%)

Table S7. Selected TDDFT singlet transitions for complex **3**.

Transition Number	Energy (eV)	Wavelength (nm)	Oscillator Strength	Major Contributions
1	284	437	0.0018	HOMO→L+1 (32%), HOMO→L+2 (27%)
2	3.02	410	0.0061	H−1→L+1 (21%), H−1→L+2 (15%), H−1→L+3 (24%)
3	3.09	402	0.0005	HOMO→L+3 (50%)
4	3.18	390	0.0005	H−3→L+2 (12%), H−3→L+3 (43%), H−1→L+3 (11%)
5	3.40	365	0.0012	HOMO→LUMO (83%)
6	3.55	349	0.0382	H−3→LUMO (10%), H−1→LUMO (64%)
8	3.72	333	0.0205	H−3→L+1 (13%), H−3→L+2 (10%), H−3→L+3 (14%), H−1→LUMO (19%), H−1→L+1 (10%)
9	3.98	312	0.0249	H−3→LUMO (72%)
11	4.17	298	0.0212	H−1→L+1 (44%), H−1→L+2 (50%)
12	4.22	294	0.1584	H−2→LUMO (74%), HOMO→L+4 (15%)
13	4.38	283	0.0233	HOMO→L+5 (61%), HOMO→L+7 (12%)
15	4.51	274	0.0583	H−2→L+1 (12%), HOMO→L+4 (47%)
17	4.57	271	0.0234	H−3→L+2 (11%), H−2→L+1 (37%), H−2→L+2 (19%)
18	4.57	271	0.0551	H−3→L+1 (23%), H−3→L+2 (16%), H−1→L+4 (24%)
20	4.63	268	0.0507	HOMO→L+6 (35%), HOMO→L+8 (36%)
21	4.66	266	0.0181	H−4→L+1 (14%), H−4→L+2 (13%), H−1→L+6 (18%), H−1→L+7 (18%), HOMO→L+8 (10%)
23	4.70	264	0.0212	H−4→L+1 (23%), H−4→L+2 (19%)
24	4.73	262	0.0242	H−1→L+6 (15%), H−1→L+8 (29%), HOMO→L+7 (16%), HOMO→L+8 (12%)
27	4.81	258	0.0902	H−1→L+6 (33%), H−1→L+7 (31%), H−1→L+8 (18%)
29	4.92	252	0.0302	H−3→L+4 (11%), H−3→L+5 (54%), H−3→L+7 (13%)

Table S8. Selected TDDFT singlet transitions for complex **4**.

Transition Number	Energy (eV)	Wavelength (nm)	Oscillator Strength	Major Contributions
1	2.84	437	0.0017	HOMO→L+2 (58%)
2	3.03	409	0.0052	H−1→L+2 (26%), H−1→L+3 (36%)
3	3.08	403	0.0004H	H−1→L+2 (19%), HOMO→L+2 (14%), HOMO→L+3 (46%)
4	3.15	393	0.0006	H−3→L+2 (25%), H−3→L+3 (30%)
5	3.39	365	0.0020	HOMO→LUMO (84%)
6	3.54	350	0.0438	H−1→LUMO (71%)
9	3.69	336	0.0131	H−3→L+3 (18%), H−1→LUMO (10%), H−1→L+1 (14%), HOMO→L+1 (10%), HOMO→L+3 (13%)
10	3.86	321	0.0655	H−1→L+1 (69%)
11	3.97	312	0.0251	H−3→LUMO (69%), H−2→LUMO (11%)
12	4.22	293	0.1589	H−3→LUMO (10%), H−2→LUMO (63%), HOMO→L+4 (16%)
13	4.26	291	0.0826	H−3→L+1 (73%), H−2→L+1 (13%)
15	4.41	281	0.0121	HOMO→L+4 (19%), HOMO→L+5 (20%), HOMO→L+7 (22%)
17	4.51	275	0.0778	H−2→L+2 (12%), HOMO→L+4 (51%)
18	4.55	273	0.0643	H−1→L+4 (54%), HOMO→L+5 (12%)
19	4.56	272	0.0234	H−2→L+2 (60%)
20	4.60	270	0.0131	H−1→L+4 (25%), H−1→L+5 (15%), HOMO→L+5 (23%)
21	4.61	269	0.0336	H−1→L+5 (15%), HOMO→L+8 (39%)
23	4.72	263	0.0273	H−2→L+3 (12%), H−1→L+8 (28%), HOMO→L+5 (11%), HOMO→L+6 (10%), HOMO→L+8 (13%)
25	4.80	259	0.0699	H−1→L+5 (31%), H−1→L+6 (20%), H−1→L+7 (10%), H−1→L+8 (18%)
26	4.86	255	0.0673	H−4→L+1 (59%)

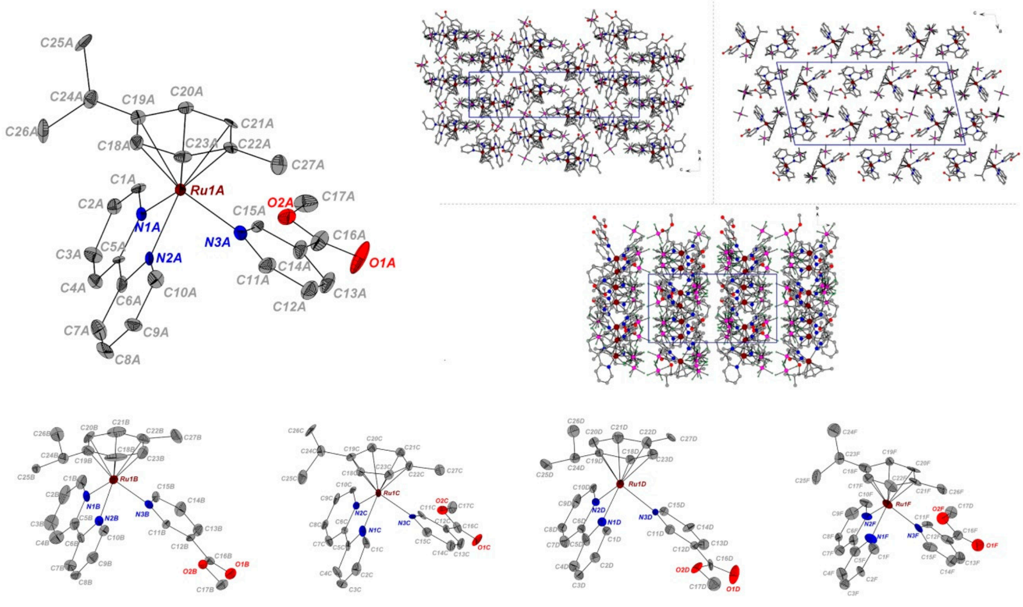

Figure S1. X-ray structures present in the asymmetric unit of [(p-cym)Ru(bpy)(*m*-COOCH$_3$-Py)][(PF)$_6$]$_2$ (**3**) and its crystal packing. Thermal ellipsoids are depicted at the 50% probability level.

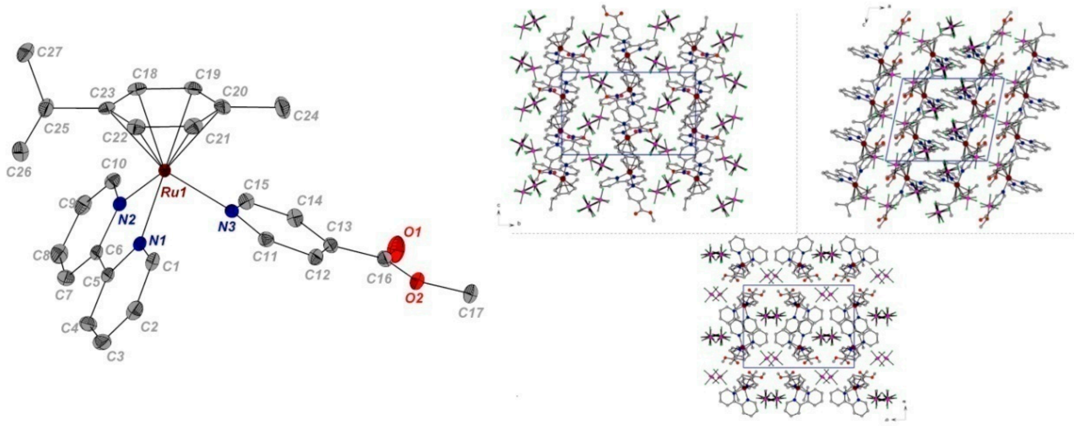

Figure S2. X-ray structure present in the asymmetric unit of [(p-cym)Ru(bpy)(*p*-$COOCH_3$-Py)][$(PF)_6$]$_2$ (**4**) and its crystal packing. Thermal ellipsoids are depicted at the 50% probability level.

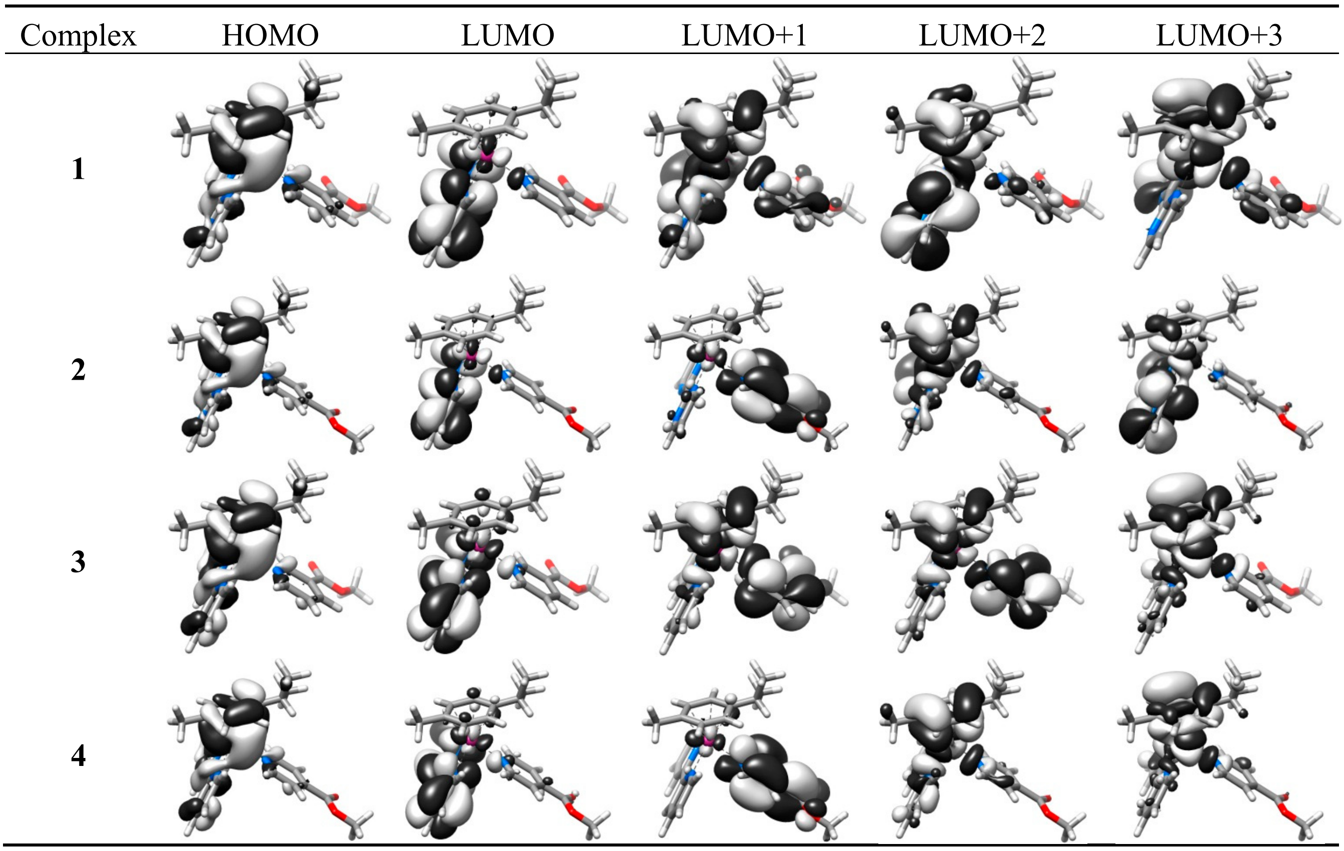

Figure S3. Selected frontier orbitals for complexes **1**–**4** in the ground state (S0) geometry.

Complex	Lowest-SOMO	Highest-SOMO
**1**	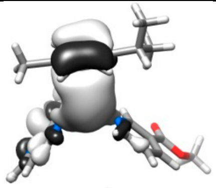	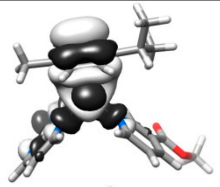
**2**	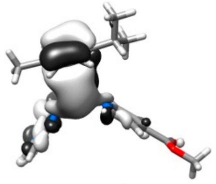	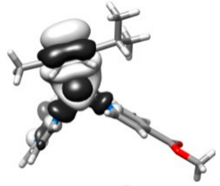
**3**	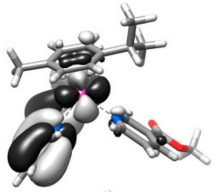	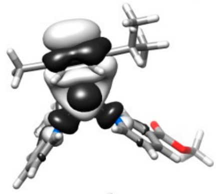
**4**	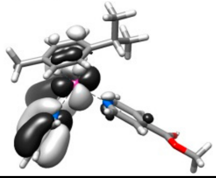	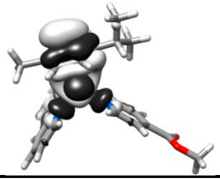

Figure S4. Calculated lowest- and highest-Single Occupied Molecular Orbitals (h-SOMO and l-SOMO, respectively, isovalue 0.02), for complexes **1**–**4** in the lowest-lying triplet (T0) optimized geometry.

Complex	Lowest-SOMO	Highest-SOMO
**1**	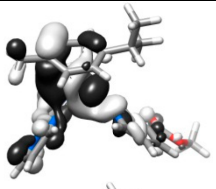	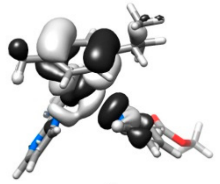
**2**	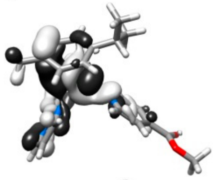	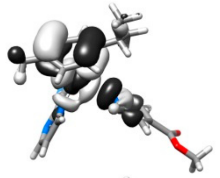
**3**	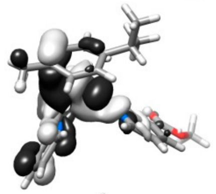	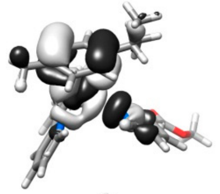
**4**	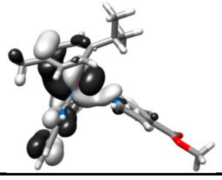	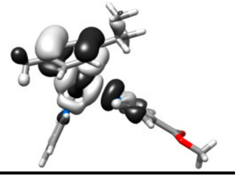

Figure S5. Calculated lowest- and highest-Single Occupied Molecular Orbitals (h-SOMO and l-SOMO, respectively, isovalue 0.02), for complexes **1**–**4** in the T1 optimized geometry.

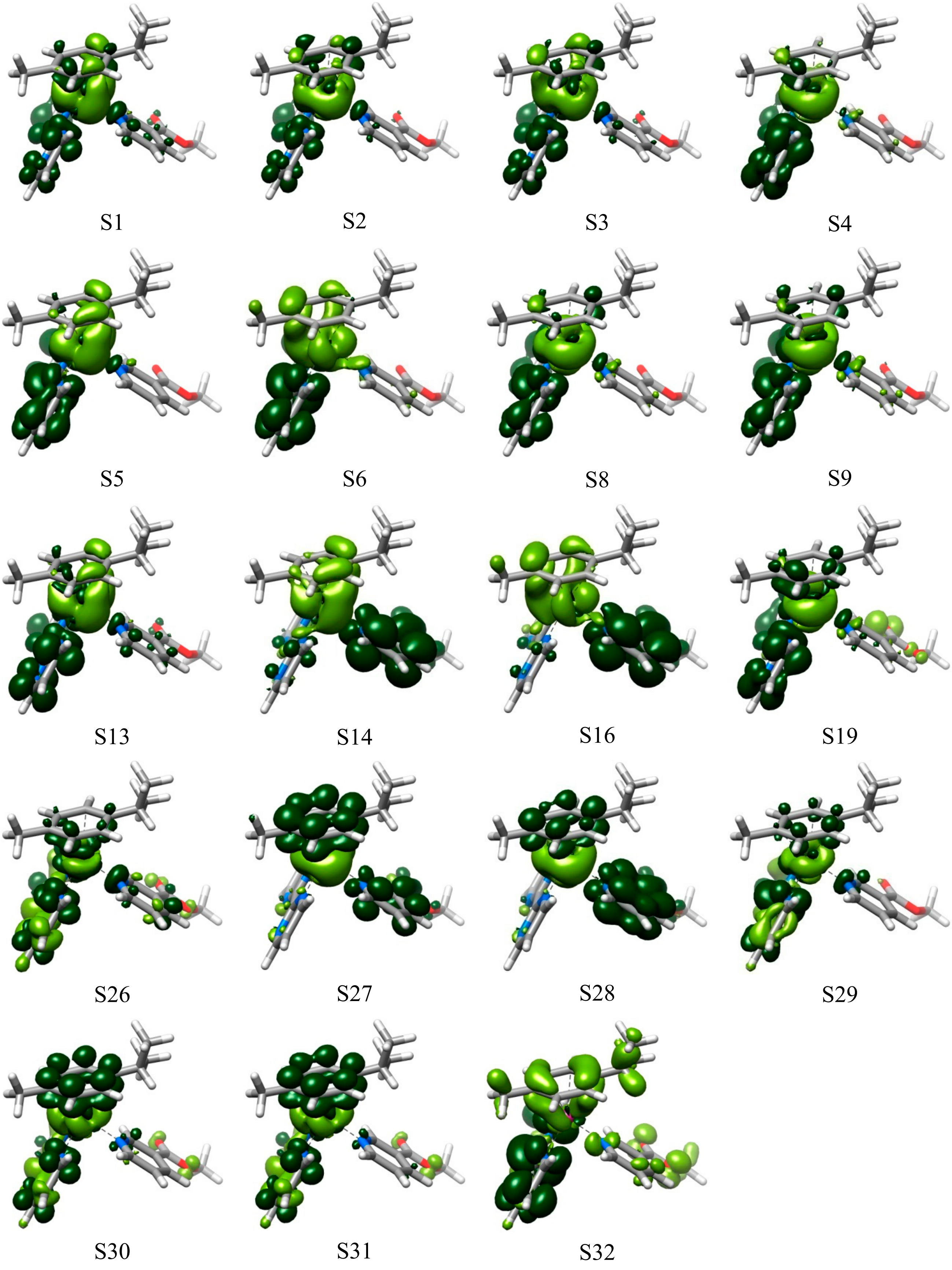

Figure S6. Selected Electron Difference Density Maps (EDDMs) of singlet excited state transitions of **1** in H_2O (light green indicates a decrease in electron density, while green indicates an increase).

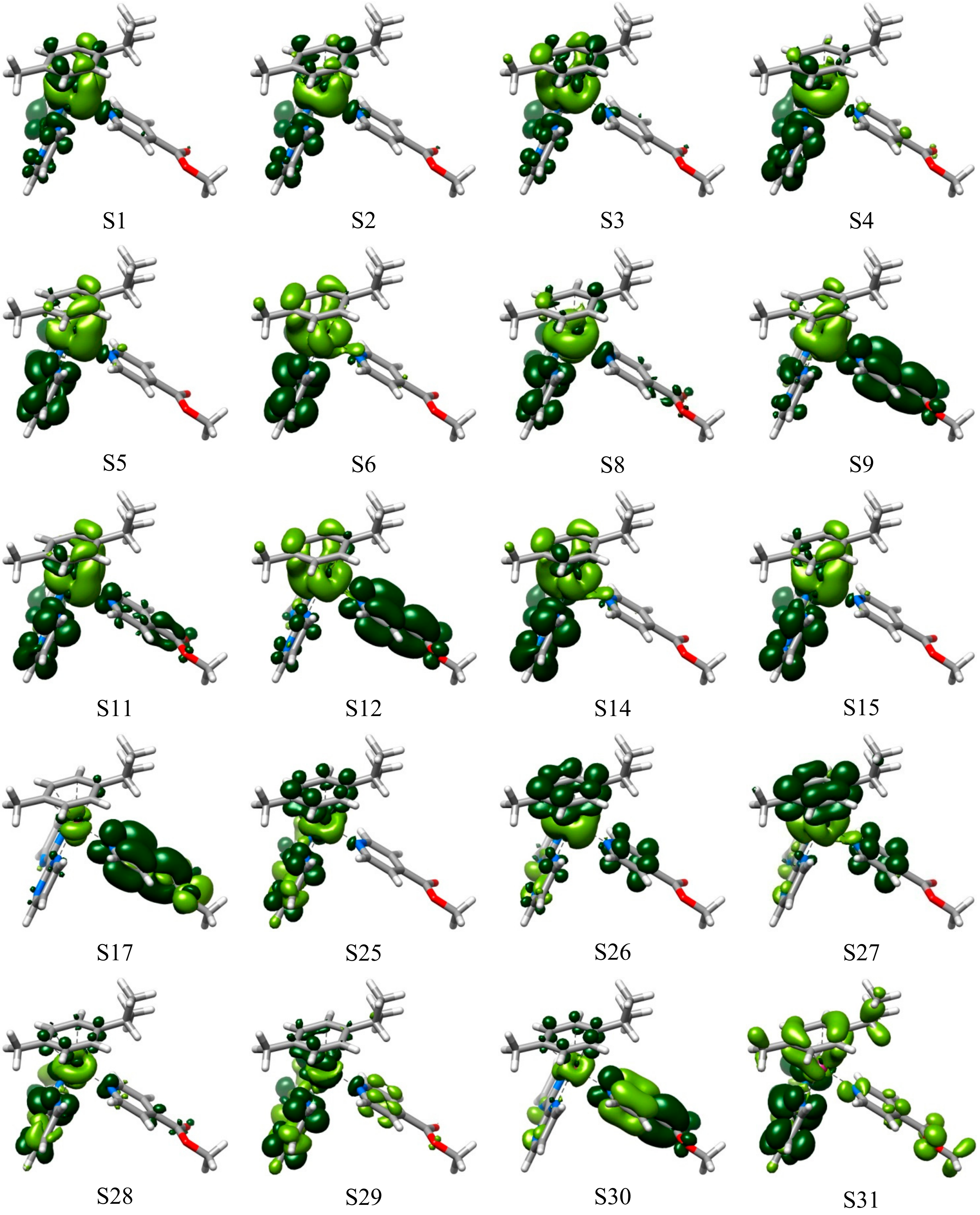

Figure S7. Selected Electron Difference Density Maps (EDDMs) of singlet excited state transitions of **2** in H_2O (light green indicates a decrease in electron density, while green indicates an increase).

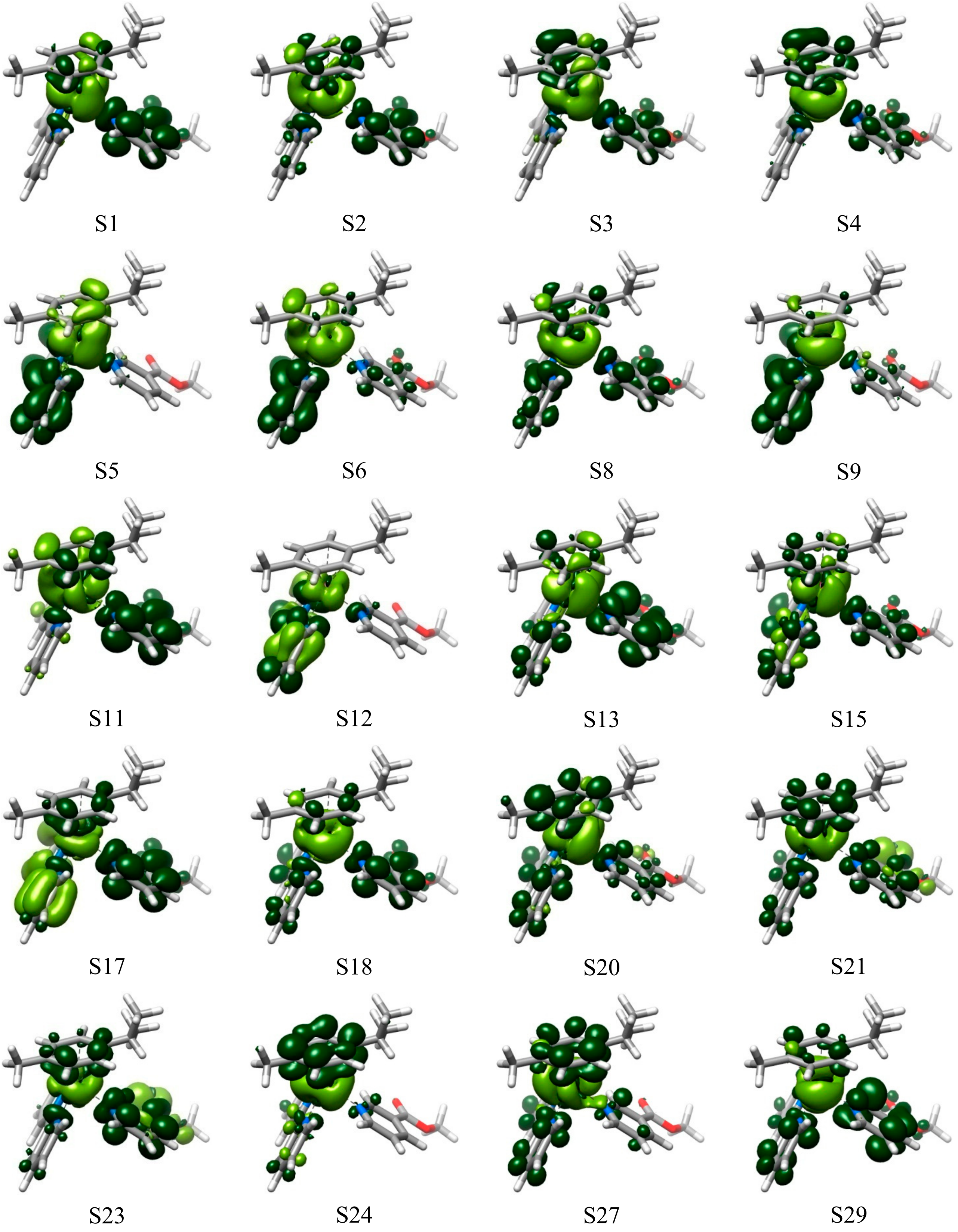

Figure S8. Selected Electron Difference Density Maps (EDDMs) of singlet excited state transitions of **3** in H_2O (light green indicates a decrease in electron density, while green indicates an increase).

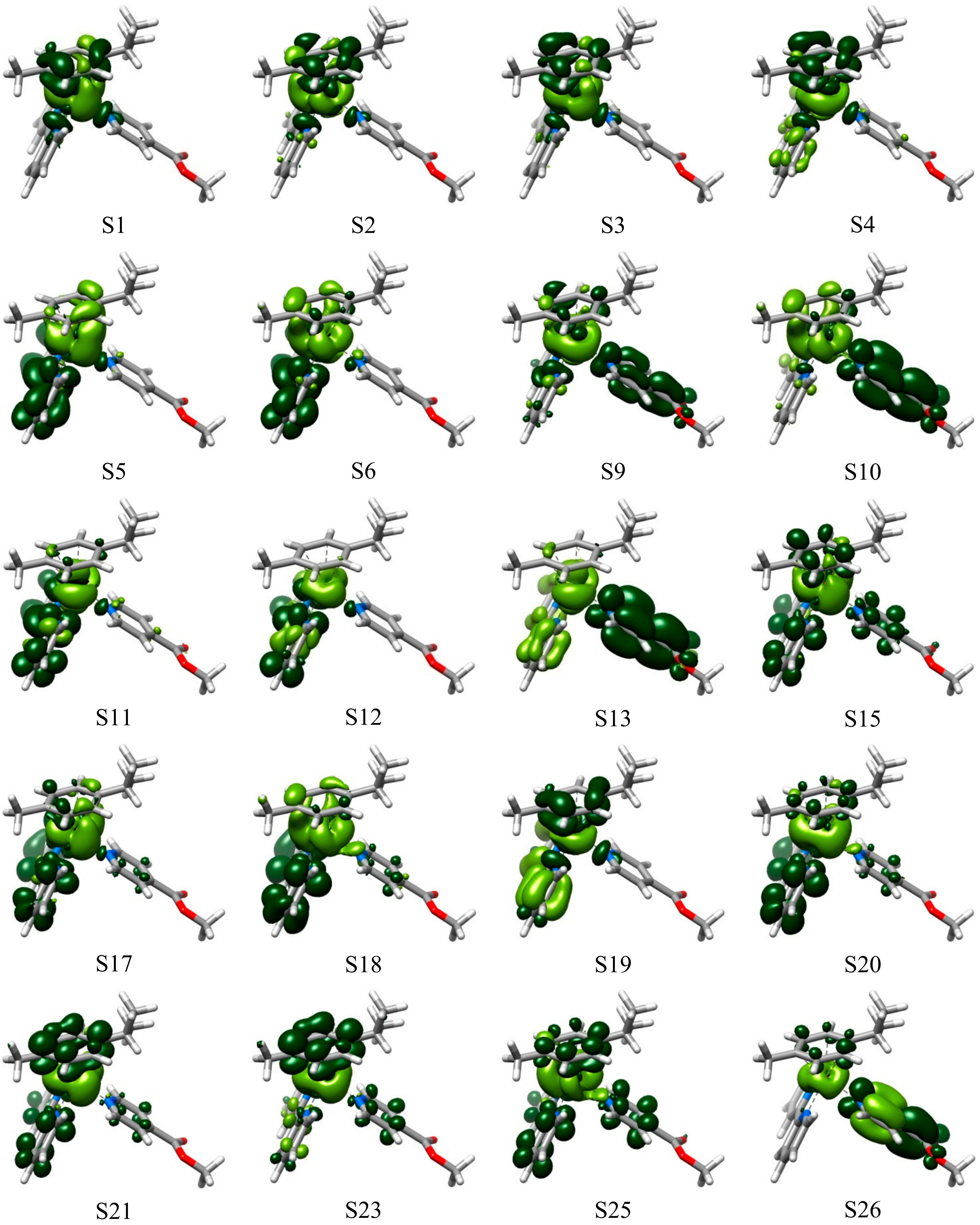

Figure S9. Selected Electron Difference Density Maps (EDDMs) of singlet excited state transitions of **4** in H_2O (light green indicates a decrease in electron density, while green indicates an increase).

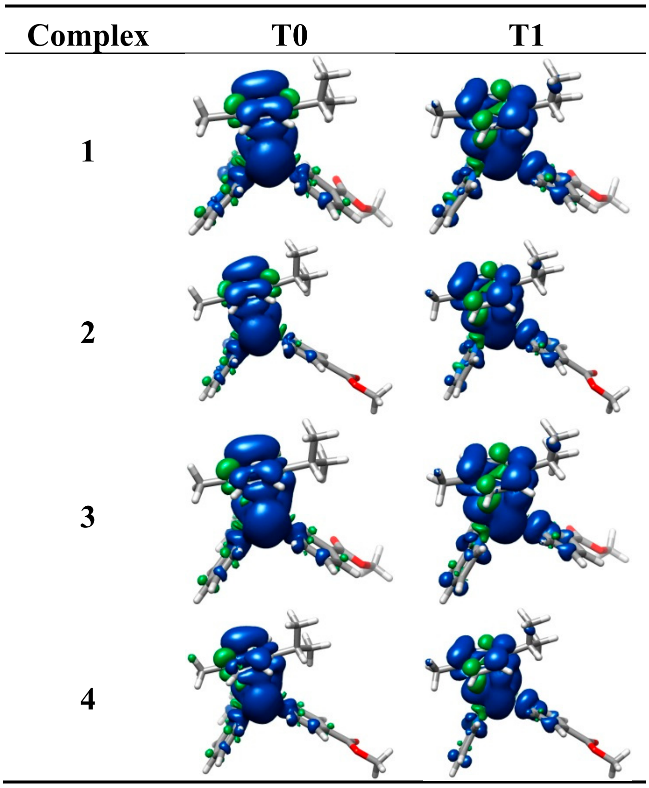

Figure S10. Calculated Spin Density Surfaces for complexes **1**–**4** in the T0 and T1 triplet optimized geometry.

Photosubstitution Quantum Yield

The quantum yields (Φ) for photoinduced ligand exchange of **1**–**4** were determined by irradiation with 395 nm light, using a MWLLS-11 Fiber Coupled 11 LED Multi-Wavelength LED Light Source (15.1 $mW \cdot cm^{-2}$) by Prizmatix Ltd. (Givat-Shmuel, Israel).

^{1}H-NMR spectroscopy of **1**–**4** in in aqueous solution (5% DMSO) was used to quantitate the decrease of reactant concentration as a function of irradiation time (moles reacted/min), and the ferrioxalate actinometer was used to determine the intensity (Einsteins/min) of the LED at 395 nm [1].

Due to the low extinction coefficients and low solubility of the complexes, optically dense (Abs > 4) solutions could not be prepared for quantum yield determinations, so the mean fraction of light (F) absorbed by the complex solution was evaluated for each compound.

In order to avoid complications from absorption of the photons by product being formed, the quantum yield measurements were conducted such that <10% conversion to product took place at each wavelength, corresponding to an irradiation time of 5 min.

Table S9. Actinometry parameters and results obtained for complexes **1**–**4**.

Sample	Reaction Rate (nmol/min)	Photon Flux (µmol/min)	F [a]	Quantum Yield
**1**	1.460	3.46	0.2200	0.0019
**2**	0.4376	3.46	0.1360	0.0009
**3**	0.2413	3.46	0.0664	0.0011
**4**	0.5781	3.46	0.0677	0.0025

[a] F is the mean fraction of light absorbed by the complexes solution.

1. Montalti, M.; Credi, A.; Prodi, L.; Gandolfi, M.T. *Handbook of Photochemistry*, 3rd ed.; CRC Press: Boca Raton, FL, USA, 2006; pp. 601−616.
